# Early loosening and secondary dislocation due to a broken trochanteric osteotomy wire following a Charnley total hip arthroplasty: a case report

**DOI:** 10.1186/1757-1626-2-7117

**Published:** 2009-05-29

**Authors:** Yousef Shahin, Rakesh Choudhary, Saeed Al-Naser, Mark Mullins

**Affiliations:** Department of Trauma and Orthopaedics Morriston HospitalHeol Maes Eglwys, Morriston, Swansea, SA6 6NLUnited Kingdom

## Abstract

We report a case of interposition of a broken trochanteric wire in the hip joint. This caused early wear of the prosthesis and dislocation of the Charnley total hip arthroplasty. The patient was treated with a revision total hip arthroplasty. This rare complication should be taken into consideration when performing a trochanteric osteotomy fixation with wiring in Charnley total hip arthroplasty.

## Introduction

Trochanteric osteotomy is a known method used in Charnley total hip arthroplasty. The advantage of the transtrochanteric approach is that it gives a better view of the hip joint. The disadvantages of this method are increased operative time, increased blood loss, trochanteric non union and trochanteric bursitis [1,2]. We report a rare complication associated with trochanteric osteotomy.

## Case presentation

**Figure 1.**
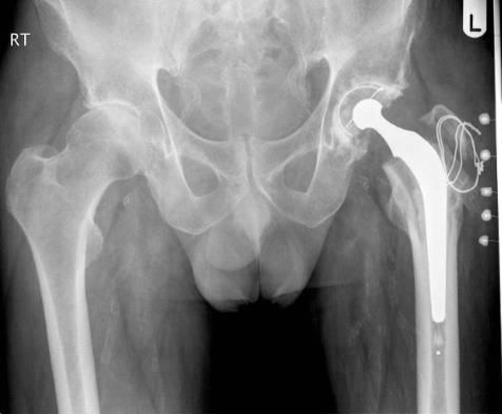
Post Charnley left total hip replacement plain X-ray of the pelvis.

A 61 year old British white male was admitted with a dislocation of a left Charnley total hip arthroplasty which was performed in 2005 for a subcapital fracture of the left neck of femur. The operation was successful for three years and the patient was doing well. Immediate post operative plain X-ray of the pelvis was satisfactory (Figure 1). In March 2008 the patient presented with a sudden left hip pain while was getting into bed and was unable to weight bear afterwards. On examination, the left leg was shortened and externally rotated.

**Figure 2.**
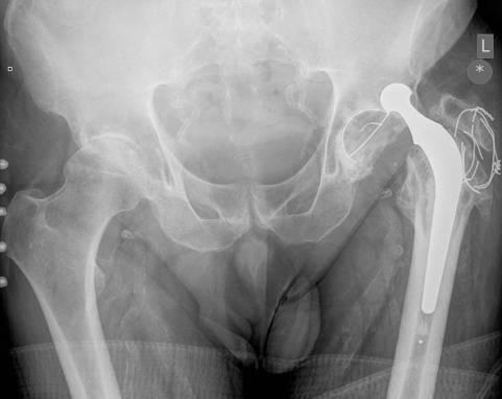
Plain X-ray of the pelvis showing a dislocated left hip with a broken wire in the left hip.

Plain X-ray of the pelvis showed a dislocated left Charnley total hip arthroplasty and it also showed a foreign body in the hip joint (broken wire) (Figure 2). The hip was reduced under general anaesthesia. The hip joint was stable up to 90 degrees flexion and 10 degrees of internal rotation.

**Figure 3.**
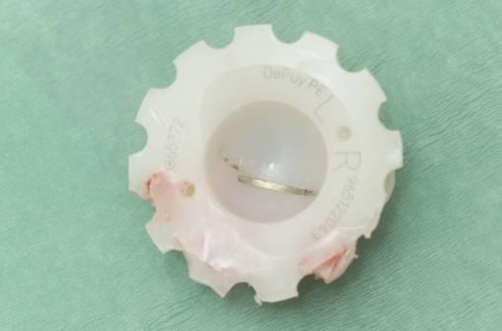
The acetabular component with a worn bearing surface and the broken wire embedded in it.

In April 2008, the patient had a revision of left Charnley total hip arthroplasty with a good result. Intraoperatively, a broken wire was found embedded in the polyethylene acetabular component and the polyethylene bearing surface was found to be worn (Figure 3).

## Discussion

Articular interposition of a broken wire in the hip joint is a rare complication following Charnley total hip arthroplasty. Four cases have been reported in the literature. The first case was a 53 year old male who had a revision arthroplasty because of a worn acetabular cup due to an intra-articular wire fragments found two years after Charnley total hip arthroplasty [3].

The second case was a 64 year old male who was found to have a wire on the X-rays after three years of the operation. He did not have any symptoms but after 12 years required revision arthroplasty due to cup wear [4].

The third case was an 80 year old female who experienced multiple dislocations of the hip 10 years following Charnley total hip arthroplasty. The wires were taken out arthroscopically [5].

The fourth case was an 80 year old female who had recurrent dislocations 10 years after the operation. Plain X-rays showed intra-articular wires. She required a revision arthroplasty due to prosthetic components wear [6].

In our case, the patient did not have any problems post operatively for three years until he experienced his first dislocation.

## Conclusion

The interposition of broken trochanteric wires in the hip joint post Charnley total hip arthroplasty can cause a third body effect leading to early loosening and early wear of the prosthetic components leading to dislocation of the hip. It is a complication that should be considered when performing a Charnley total hip arthroplasty which might necessitate an early revision surgery.
